# Supplementary material for: Blood meal-induced inhibition of vector-borne disease by transgenic microbiota
Source: Nat Commun. 2018 Oct 8;9:4127. doi: 10.1038/s41467-018-06580-9 (PMC6175951; doi:10.1038/s41467-018-06580-9)
Supplement: Supplementary file 1 — Supplementary Information [file 41467_2018_6580_MOESM1_ESM.pdf]

Blood meal-induced inhibition of vector-borne disease by transgenic microbiota

Shane et al.

Supplementary information

Supplementary figures

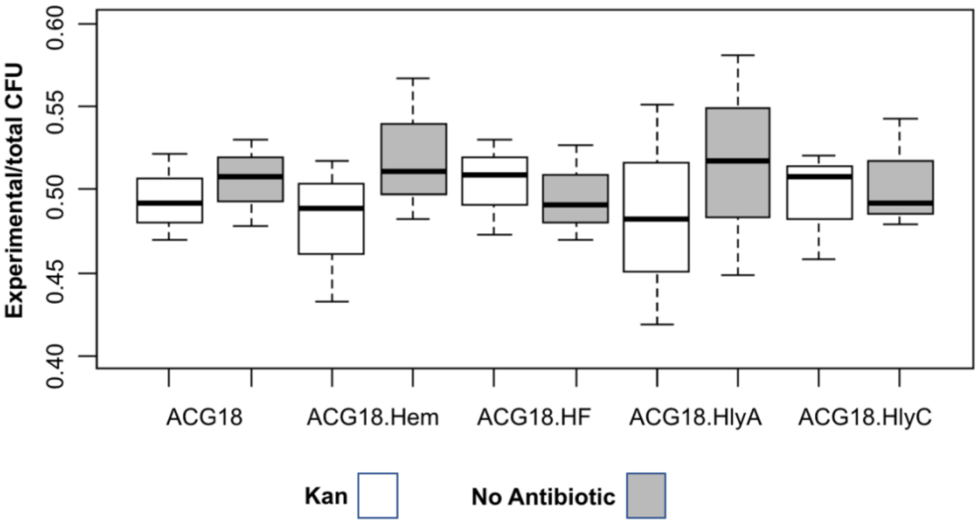

Supplementary Figure 1 Antiplasmodial *Asaia* strains retain plasmids at least 6 h without antibiotic selection. Three individual colonies were tested for each strain. They were grown in minimal media with kanamycin to log phase, then washed twice, and resuspended in minimal media to OD_{600} =0.50. These cultures were grown for 6 h then plated with and without antibiotic selection. The mean number of CFUs were compared for each strain under each condition. No significant difference between the growth conditions for any strain was detected (Welch's t-test, P>0.05). Box bars are medians. The top and bottom of the boxes represent the first and third quartile of the data spread. The lower and upper bounds of the whiskers are the lowest datum still within 1.5 X interquartile range (IQR) of the lower quartile, and the highest datum still within 1.5 X IQR of the upper quartile, respectively.

Supplementary figure 2

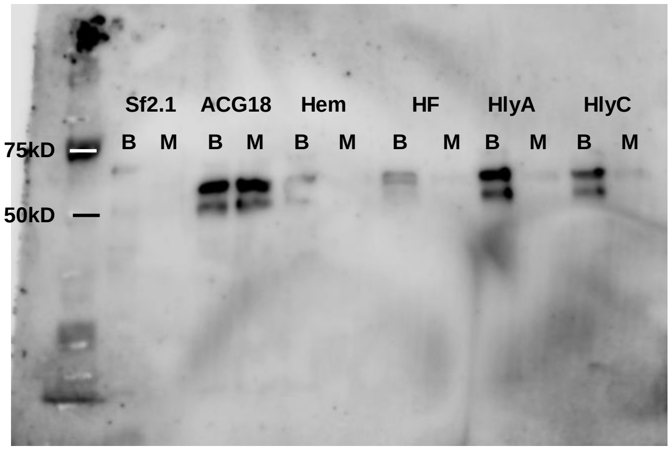

Supplementary Figure 2 Original uncropped western blot corresponding to figure 4 in the manuscript, marked in the same manner.

Supplementary Table 1 Bacterial species and plasmids used in this study		
**Species**	**Relevant Characteristics**	**Source**
E. coli Top 10 F'	F⁻ *mcrA* Δ(*mrr-hsdRMS-mcrBC*) *Φ80lacZ*ΔM15 Δ*lacX74 recA1 araD139* Δ(*ara-leu*)7697 *galU galK rpsL* (Str^z) *endA1 nupG*	1
Asaia bogorensis	Isolate from orchid tree flower, Indonesia (ATCC BAA-21)	2
Asaia sp. SF2.1	Wild-type strain isolated from *Anopheles* mosquitoes	3
**Plasmids**	**Relevant Characteristics**	**Source**
pGLR1	Promoterless GFP-lux Dual reporter, Kanamycin resistance, pBBR origin. Creates *Asaia* strain AGLR1.	4
pGLR1. Hem	pGLR1 with 500 base pair HmuT homolog promoter at MCS for GFP-lux dual reporter. Creates *Asaia* strain AGLR1.Hem.	This study
pGLR1.HF	pGLR1 with shortened HmuT homolog promoter at MCS for GFP-lux dual reporter. Creates *Asaia* strain AGLR1.HF.	This study
pGLR1.SodB	pGLR1 with SodB homolog promoter at MCS for GFP-lux dual reporter. Creates *Asaia* strain AGLR1.SodB.	This study
pGLR1.AcnA	pGLR1 with AcnA homolog promoter at MCS for GFP-lux dual reporter. Creates *Asaia* strain AGLR1.AcnA.	This study
pGLR1.Bfr	pGLR1 with Bfr homolog promoter at MCS for GFP-lux dual reporter. Creates *Asaia* strain AGLR1.Bfr.	This study
pGLR1.Ferr	pGLR1 with ThxC homolog promoter at MCS for GFP-lux dual reporter. Creates *Asaia* strain AGLR1.Ferr.	This study
pGLR1.HlyA	pGLR1 with HlyA homolog promoter at MCS for GFP-lux dual reporter. Creates *Asaia* strain AGLR1.HlyA.	This study
pGLR1.HlyC	pGLR1 with HlyC homolog promoter at MCS for GFP-lux dual reporter. Creates *Asaia* strain AGLR1.HlyC.	This study
pBBR1MCS-2	Used for plasmid construction	5
pNB50	Constitutive GFP plasmid driven by the *nptII* promoter. Creates *Asaia* strain ANB50.	6
pNB92	P_{nptII} driving expression of '*phoA*. No signal sequence, pBBR1 backbone, KanR	7
pNB97	P_{nptII} driving expression of scorpine-*phoA*. pBBR1 backbone, KanR. Source of scorpine for pCG18.	7
pCG6	pNB92 containing the *Asaia* TonB-dependent protein 1 secretion signal.	This study
pCG18	Antimalarial Scorpine driven by *nptII* promoter, TonB secretion signal, PhoA reporter gene, Kanamycin resistance. Creates *Asaia* strain ACG18.	This study
pCG18.glr1	pCG18 with pGLR1 terminator and MCS upstream of scorpine. Creates *Asaia* strain ACG18.glr1.	This study
pCG18.Hem	pCG18.glr1 with 500 bp HmuT homolog promoter at MCS driving scorpine. Creates *Asaia* strain ACG18.Hem.	This study
pCG18.HF	pCG18.glr1 with shortened HmuT homolog promoter at MCS driving scorpine. Creates *Asaia* strain ACG18.HF.	This study
pCG18.HlyA	pCG18.glr1 with HlyA homolog promoter at MCS driving scorpine. Creates *Asaia* strain ACG18.HlyA.	This study
pCG18.HlyC	pCG18.glr1 with HlyC homolog promoter at MCS driving scorpine. Creates *Asaia* strain ACG18.HlyC.	This study

Supplementary Table 2 Primers and other synthetic DNA used in this study		
	**Sequence 5' - 3'**	**Purpose**
FAsodB.AVRII	TACCTAGGgtcatcaacggcatcatgggc	Cloning promoter region of *sodB* into pGLR1
RAsodBSPHI	TAgcatgcggtcgcgtctcctggact	Cloning promoter region of *sodB* into pGLR1
FAacnaAVRII	TACCTAGGatccagcgctgcaccc	Cloning promoter region of *acnA* into pGLR1
RAacnaSPHI	TAgcatgcggcttgtcctcctgccttg	Cloning promoter region of *acnA* into pGLR1
FAbfrAVRII	TACCTAGGccagcgtgatcgagagtgg	Cloning promoter region of *bfr* into pGLR1
RAbfrSPHI	TAgcatgccagccattccacttcagaacgg	Cloning promoter region of *bfr* into pGLR1
FAhemAVRII	TACCTAGGggtgaggatcatgtagggct	Cloning promoter region of *hmuT* into pGLR1
RAhemSPHI	TAGCATGCggttcctgacccttttctgc	Cloning promoter region of *hmuT* into pGLR1
FAferrEcoRI	GAATTCctctccggttgccc	Cloning promoter region of *trxR* homolog into pGLR1
RAferrBamHI1	GGATCCccatatctgtccgg	Cloning promoter region of *trxR* homolog into pGLR1
FAhlyaAvrII	TACCTAGGcgcccgatcttgaaac	Cloning promoter region of *hlyA* into pGLR1
RAhlyaSphI	TAGCATGCtggggcttttccgc	Cloning promoter region of *hlyA* into pGLR1
FpGLR1term/mcsNsiI	TACAGTATGCATggcgcgcccagctg	Cloning of terminator and MCS of pGLR1 into pCG18 at promoter region
RpGLR1term/mcsNdeI	TACAGTCATATGtttttcctcctgcatgcc	Cloning of terminator and MCS of pGLR1 into pCG18 at promoter region
FpGLR1term/mcsGibs	TTGTGATGGCTTCCATGTCGcagctgtctagggcggcgg	Gibson assembly of pGLR1 construct promoter regions into pCG18
F18promGibs	GAAAAACATATGAAACTTCGCAAG	Gibson assembly of pGLR1 construct promoter regions into pCG18
R18promGibs	cgacatggaagccatcac	Gibson assembly of pGLR1 construct promoter regions into pCG18
RGLRhfGibs	CTGCTTGCGAAGCTTCATATGTTTTTCCctaatgctcatggttcctg	Gibson assembly of pGLR1.HF promoter regions into pCG18
RGLRhlyaGibs	CGAAGCTTCATATGTTTTTCgcatgccaagcccaagggtg	Gibson assembly of pGLR1.HlyA promoter regions into pCG18

TonB-dependent receptor protein 1 singal sequence G-block	tttgaacCATATGaagcttcgcaagcagcgccatgcccttacgctgtctctcttctgctctcccctcgccctgctgagcgccaccgctcacgccgcagatgcgaagcttcccgttcatcacaatacggcacccgtttcgcagctgcatgcccagaacgctTTAATTAAgacttc	A dsDNA fragment used to clone the first 150 nucleotides of the TonB-dependent receptor protein 1 into pCG18

Supplementary references

1. Durfee, T. *et al.* The complete genome sequence of Escherichia coli DH10B: Insights into the biology of a laboratory workhorse. *J. Bacteriol.* **190,** 2597–2606 (2008).

2. Yamada, Y. *et al.* Asaia bogorensis gen. nov., sp. nov., an unusual acetic acid bacterium in the alpha-Proteobacteria. *Int J Syst Evol Microbiol* **50 Pt 2,** 823–829 (2000).

3. Favia, G. *et al.* Bacteria of the genus Asaia stably associate with Anopheles stephensi, an Asian malarial mosquito vector. *Proc. Natl. Acad. Sci.* **104,** 9047–9051 (2007).

4. Benedetti, I. M., de Lorenzo, V. & Silva-Rocha, R. Quantitative, non-disruptive monitoring of transcription in single cells with a broad-host range GFP-luxCDABE dual reporter system. *PLoS One* **7,** e52000 (2012) doi: 10.1371/journal.pone.0052000.

5. Kovach, M. E. *et al.* Four new derivatives of the broad-host-range cloning vector pBBR1MCS, carrying different antibiotic-resistance cassettes. *Gene* **166,** 175–176 (1995).

6. Bongio, N. J. Secretion of Malaria Transmission-Blocking Proteins from Paratransgenic Bacteria. PhD Thesis. Duquesne University, 2015).

7. Bongio, N. J. & Lampe, D. J. Inhibition of Plasmodium berghei development in mosquitoes by effector proteins secreted from Asaia sp. Bacteria using a novel native secretion signal. *PLoS One* **10,** e0143541 (2015) doi: 10.1371/journal.pone.0143541.
